# Supplementary material for: A pipeline for cell migration analysis in live-cell imaging data from human iPSC-derived forebrain assembloids
Source: Front Cell Dev Biol. 2026 Jul 1;14:1880548. doi: 10.3389/fcell.2026.1880548 (PMC13368796; doi:10.3389/fcell.2026.1880548)
Supplement: Supplementary file 1 [file DataSheet1.PDF]

Data supplement

A pipeline for cell migration analysis in live-cell imaging data from human iPSC-derived forebrain assembloids.

Maya P. Weidman[1], Natalie Baker Campbell[2#], Cody Headings[1#], Samantha Chung[2], Musarat Khan[1], Aarnav Kandukuri[1], Vianne Lim[1], Gloria Olubowale[1], Michelle Kim[2], Anna Devor[1,3,4], Ella Zeldich[2,3,5*] and Martin Thunemann[1,3*]

1. Department of Biomedical Engineering, Boston University, Boston, MA, USA
2. Department of Anatomy & Neurobiology, Boston University Chobanian & Avedisian School of Medicine, Boston University, Boston, MA, USA
3. Neurophotonics Center, Boston University, Boston, MA, USA
4. Athinoula A. Martinos Center for Biomedical Imaging, Department of Radiology, Harvard Medical School, Massachusetts General Hospital, Charlestown, MA, USA
5. Center for Systems Neuroscience, Boston University, Boston, MA, USA

Supplementary Table 1. Estimation of signal-to-background (SBR) and signal-to-noise (SNR) ratios before and after background subtraction for EYFP- and tdTomato-labeled structures.

**EYFP**	**Before background subtraction**					**After background subtraction**				
**ROI**	**Signal**	**Back-ground**	**s.d. (back-ground)**	**SBR**	**SNR**	**Signal**	**Back-ground**	**s.d. (back-ground)**	**SBR**	**SNR**
1	184.10	6.28	125.77	1.46	29.34	189.60	4.16	3.12	60.75	45.58
2	281.15	5.92	132.98	2.11	47.52	139.15	1.76	0.63	219.48	78.88
3	280.38	5.29	123.23	2.28	53.02	147.38	1.94	1.02	144.07	75.93
4	247.19	3.24	107.49	2.30	76.41	137.19	1.76	1.35	102.00	77.82
5	175.79	3.51	106.77	1.65	50.08	69.79	2.73	2.39	29.26	25.55
6	305.64	3.41	105.60	2.89	89.66	200.64	2.55	2.16	93.02	78.56
7	155.25	3.40	106.80	1.45	45.66	48.25	2.36	1.75	27.59	20.44
8	178.50	4.27	120.62	1.48	41.85	58.50	2.78	2.00	29.25	21.08
9	291.24	6.38	132.85	2.19	45.63	155.24	3.39	1.99	77.85	45.75
10	232.78	5.96	148.48	1.57	39.08	75.78	1.83	0.62	121.63	41.45
**Mean**				**1.94**	**51.83**				**90.49**	**51.10**
	**Fold-improvement in SNR**					**0.99**				
	**Fold-improvement in SBR**					**46.68**				

**tdTomato**	**Before background subtraction**					**Bfter background subtraction**				
**ROI**	**Signal**	**Back-ground**	**s.d. (back-ground)**	**SBR**	**SNR**	**Signal**	**Back-ground**	**s.d. (back-ground)**	**SBR**	**SNR**
1	234.91	111.33	4.09	2.11	57.50	121.91	1.28	2.21	95.16	55.16
2	392.41	117.77	5.87	3.33	66.85	259.41	1.68	2.94	154.69	88.35
3	612.31	104.61	3.19	5.85	192.01	505.31	1.13	1.92	447.57	263.59
4	397.30	104.79	2.99	3.79	132.83	292.30	1.58	2.09	184.88	139.59
5	411.02	106.90	3.68	3.85	111.72	289.02	1.72	2.59	168.43	111.72
6	1467.58	247.37	22.51	5.93	65.19	1193.31	2.56	6.75	465.77	176.73
7	1575.71	279.36	38.23	5.64	41.22	1337.42	54.53	36.67	24.53	36.48
8	1988.03	239.52	39.83	8.30	49.91	1790.21	47.55	38.33	37.65	46.70
9	1773.87	160.18	14.26	11.07	124.44	1618.87	10.35	12.82	156.35	126.33
10	561.28	128.35	6.64	4.37	84.56	422.00	1.17	2.79	360.07	151.20
**Mean**				**5.43**	**92.62**				**209.51**	**119.59**
	**Fold-improvement in SNR**					**1.29**				
	**Fold-improvement in SBR**					**38.62**				

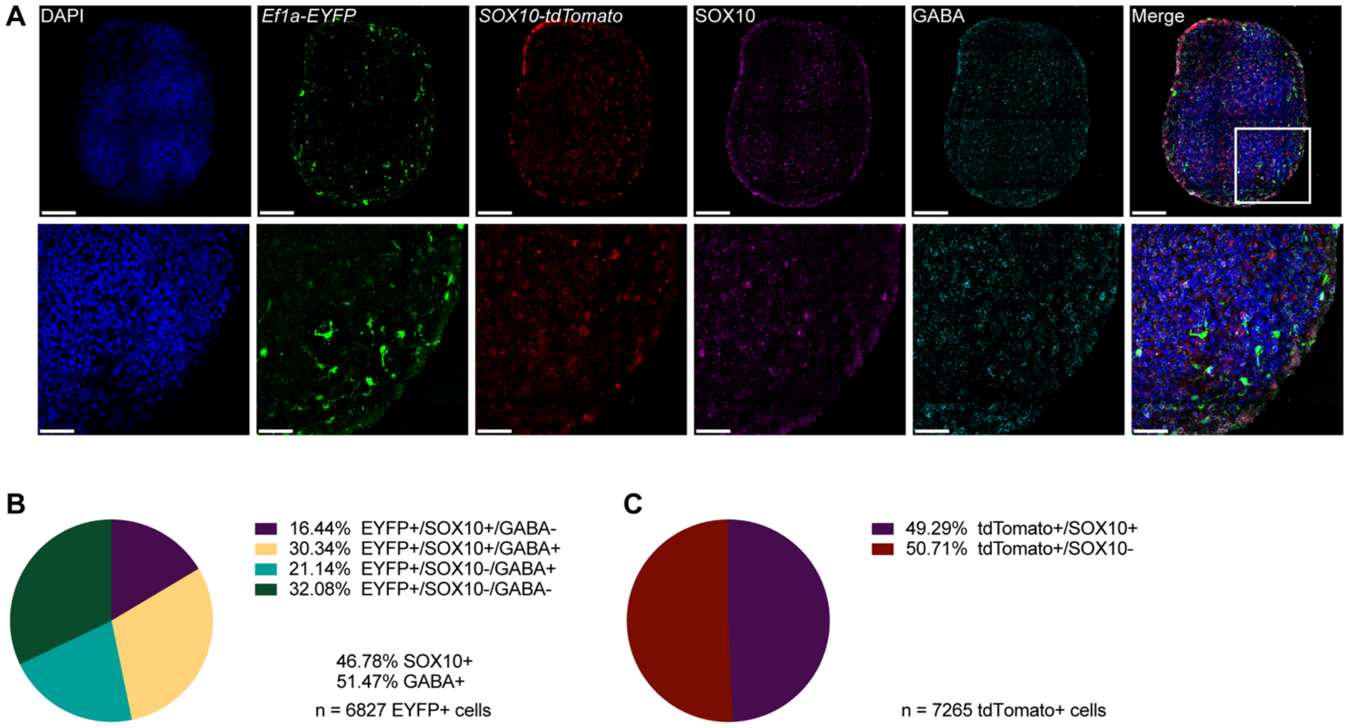

Supplemental Figure 1: Efficiency of viral labeling in vCOs. (A) Representative immunofluorescence images showing viral labeling of Ef1α-EYFP, SOX10-tdTomato, and immunohistochemical staining with SOX10, and GABA antibodies in vCOs at 82 days of differentiation. Scale bars are 150μm and 50μm (inset). (B, C) Quantification analysis was performed using QuPath across the same 3 vCOs. (B) Pie chart showing percentage breakdown of $EYFP^+$ cells colocalized with SOX10 and GABA. n = 6827 $EYFP^+$ cells. (C) Pie chart showing percentage breakdown of $tdTomato^+$ cells colocalized with SOX10. n = 7265 $tdTomato^+$ cells.

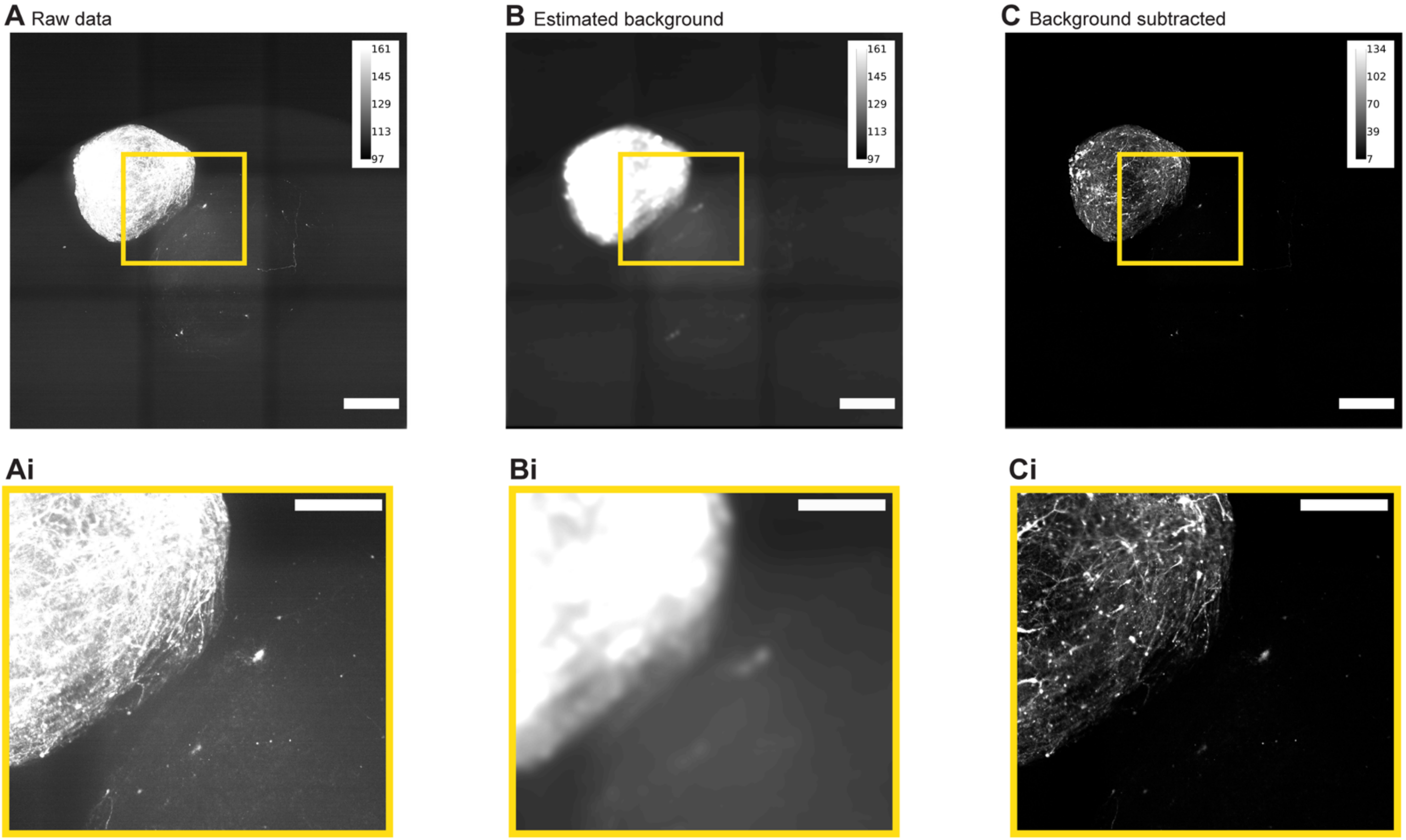

Supplemental Figure 2: Background subtraction pipeline applied to Sox10-tdTomato^{+} channel corrects signal heterogeneity. (A–C) Maximum intensity projections (z-MIP) of the same representative assembloid shown in Figure 2 at a single timepoint, imaged in the red channel: raw data (A), estimated background generated by 3D Gaussian filtering (FWHM: 64.3 × 64.3 × 424 µm) (B), and background-subtracted output (C). Intensity calibration bars are shown for each panel. Yellow boxes indicate the region shown in Ai–Ci. (Ai–Ci) Zoomed insets of the indicated region, displayed at identical brightness/contrast settings within the inset row. Background subtraction markedly reduces illumination gradients and enhances visibility of SOX10-tdTomato^{+} cell processes across the vCO and dCO regions. Signal-to-background ratio improved from 1.9 (raw) to 90.5 (corrected), approximately a 47-fold increase, measured from n = 10 SOX10-tdTomato^{+} structures using straight-line intensity profiles drawn across the identical structures in both raw and background-subtracted images in FIJI/ImageJ. The same pipeline parameters were applied without modification from the EYFP^{+} channel. Scale bars: 500 µm (A–C); 250 µm (Ai–Ci).

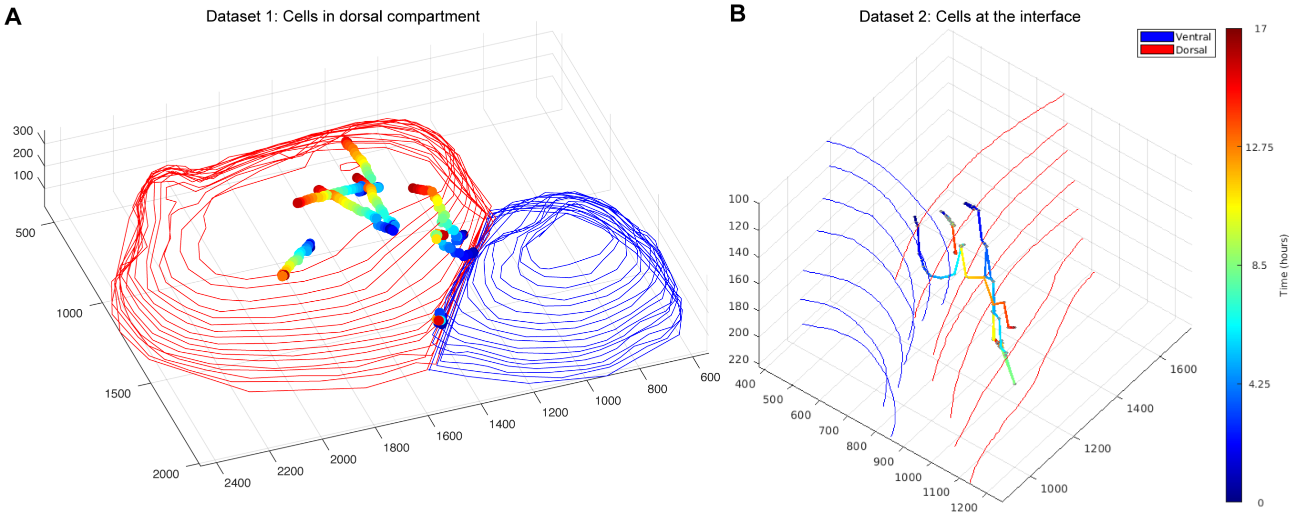

Supplementary Figure 3. Cell migration trajectories relative to the interface between dorsal and ventral part of the assembloid. (A) 3D view of Dataset 1 (corresponding to Supplementary Video 1) indicates the outlines of ventral (blue) and dorsal (red) part of the assembloid across Z planes and the trajectories of six cells. (B) 3D view of Dataset 2 highlights migration patterns of five cells that are close to or that crossed the boundary between ventral (blue) and dorsal (red) part of the assembloid within the recording period. Outlines were manually drawn on the average image across all time points as polygonal regions of interest in ImageJ/FIJI. Both panels show data from the EGFP channel. Coordinates along the image axes are given in µm.

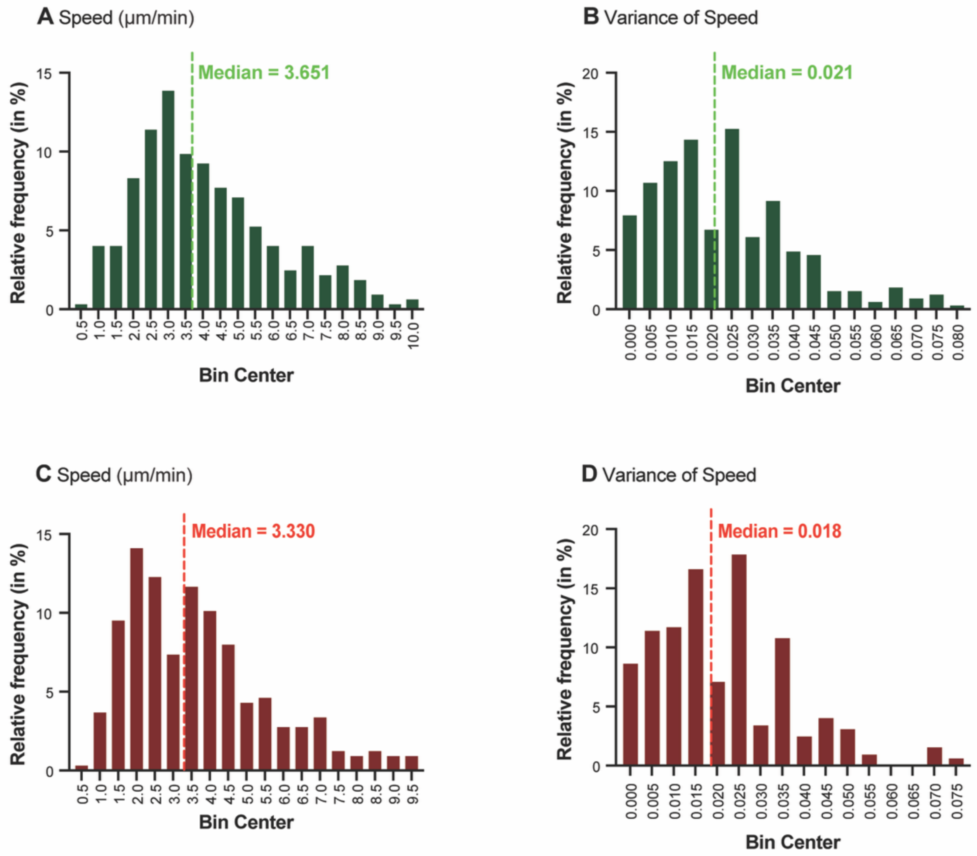

Supplemental figure 4: Speed and speed variance histograms for both EYFP$^+$ and tdTomato$^+$ tracked cell populations. (A, B): n = 336 EYFP$^+$ tracked cells. (C, D): n = 337 tdTomato$^+$ tracked cells. (A, C) Average instantaneous speed (μm/min): mean frame-to-frame speed computed as 3D Euclidean step distance divided by the 30-minute frame interval. Distributions are approximately unimodal with medians of 3.651 μm/min (EYFP$^+$) and 3.330 μm/min (tdTomato$^+$), with most cells falling between 1.5 and 6 μm/min in both channels. (B, D) Variance of speed ((μm/min)^2): variance of instantaneous frame-to-frame speeds across the full 17-hour imaging period. Markedly right-skewed distributions with medians of 0.021 (μm/min)^2 for EYFP$^+$ cells and 0.018 (μm/min)^2 tdTomato$^+$ cells, consistent with relatively steady migration paces in both populations.

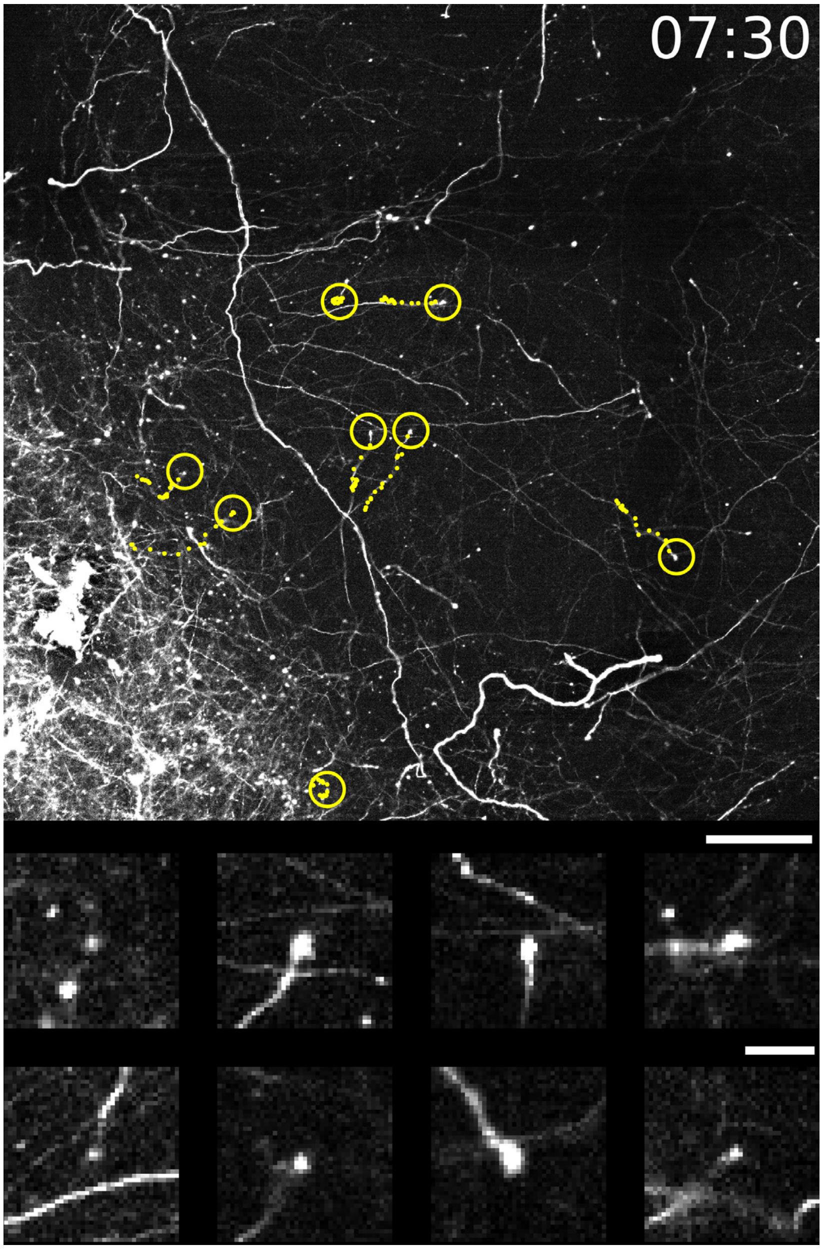

Supplementary Video 1. (Top) Time series of maximum intensity projections across the Z axis with X-Y locations of eight cells (EGFP channel); the current position is shown as open circle, and previous positions are shown as dots. The field of view is focused on a region containing the boundary between ventral (bottom left) and dorsal (top right) part of the assembloid. 3D views of the trajectories are shown in Supplementary Figure 3A. Scale bar, 200 µm. (Bottom) Eight individual time series of maximum intensity projections across the Z axis for each cell shown in the top panel. The center of the field of view is defined by the X and Y coordinates at each time point. Intensities the time series were normalized independently for each cell. Scale bar, 25 µm.
